# After All, What?!

**Published:** 1910-09

**Authors:** 


### After All, What?!

The machinery of the American Medical Association seems to be developing more and more horse or bull power, and as the revolutionary flywheel revolves the smaller medical fry seem to be more and more drawn toward the vortex that is enlarging to suck in everything suckable! It has evidently been forced upon the minds of many manufacturers and impressionable physicians that it is a good thing to "stand in" with the machine in order to avoid being put out of business; and possibly to increase a waning business!

In looking over its *Journal* for May 23rd we are struck with the number and variegated color of its advertisements and advertisers. It evidently pays to be in good company if we judge from the advertising patronage shown in this number, and while many of these advertisements convey no idea of the formula or contents, still we have the assurance of this self-righteous and all-wise publication that its chemists have looked into these matters and we can trust it all to them as being all right. Now, this is a comfort to any prescriber to know that he can give "Mergal" in syphilis, "Bravalol" in nervousness and "Neuronidia" for insomnia without question, without fear and without any knowledge of the composition save the statement of the celebrated water-cure fakir of Lincoln, Neb., and his association pals who are conjointly running the whole machinery for the easily led dupes in the medical profession.

To us we see no difference between these talismanic names as they appear either in the patent medicine admanacs or in the Journal of the American Medical Association. In fact, we have more regard for the out and out fakir than for the one who has been whitewashed by the Association but is still in need of thorough disinfection for the good of his associates, for as much as they may try to slur over the Lydstonian records, the independent medical press has as a unit shown the true character of the Ass in the Lion's Skin, who, of all others, is selected to give a clean, moral passport to the manufacturers and prescribers of this country. Some things make us sick; we don't blame the medico-political machine of Chicago, nor its manipulators for the money they make out of it, but we hate to see simple Simons caught with such bait as this crowd hands out.

We note in this same issue four columns of editorial devoted to fulsome praise of the character, personnel and laudable work of the Council on Pharmacy and Chemistry—a department of this machine. The burden of the song is that these two or three chemists have spent the money of the Association pretty freely in analyzing the proprietaries and giving the profession the *truth* concerning their composition. The inference being that all over the land the chemists and scientific workers, when engaged in preparing special remedies or proprietaries, were equally engaged in attempting to deceive the profession and defraud the public!

Now, this is a wonderful proposition to ask a sensible physician to believe, and when we take into consideration the dual role of one-time quack and one-time whitewashed reformer under which the chief promulgator of these *laboratory truths* masquerades, we would accept with equal faith the claims of the Water-Cure Institute of Lincoln, Neb., and Rupert Wells' Cancer Institute of St. Louis.—*Editorial in Southern Clinic, C. A. Bryce, M. D., Editor.*

